# 68 Screen & Intervene: Mental Health Screening in the Burn Center

**DOI:** 10.1093/jbcr/irae036.060

**Published:** 2024-04-17

**Authors:** Amanda R Alarcon, Linda Colarusso, Eli Strait, Jeanne Lee, Alan M Smith, Arpi Minassian

**Affiliations:** University of California, San Diego, San Diego, California; UC San Diego Health, San Diego, California; University of California, San Diego, San Diego, California; UC San Diego Health, San Diego, California; University of California, San Diego, San Diego, California; UC San Diego Health, San Diego, California; University of California, San Diego, San Diego, California; UC San Diego Health, San Diego, California; University of California, San Diego, San Diego, California; UC San Diego Health, San Diego, California; University of California, San Diego, San Diego, California; UC San Diego Health, San Diego, California

## Abstract

**Introduction:**

Depression, Acute Stress Disorder (ASD) and Post Traumatic Stress Disorder (PTSD) in burn patients are common during the first year of recovery and beyond. Mental health screening of patients at all burn centers is recommended. The purpose of this study was to determine how depression or PTSD symptoms may evolve from inpatient to outpatient care in burn patients.

**Methods:**

From March of 2022 through September of 2023, the PHQ-2 and PC-PTSD were administered to 280 outpatients via a self-report screening questionnaire. For pediatric patients < 12 years old, the primary caregiver filled out the questionnaire. A score of 2 or more on either of the screening tools resulted in further assessment by the social worker to determine appropriate next steps and provide community referrals. A subset of these patients were also screened in the inpatient setting via interview by the psychology team using the PHQ-2 and a modified PC-PTSD. The chi-square test was used to analyze the data collected.

**Results:**

A total of 106/280 (38%) outpatients screened positive for depressive symptoms, 110/280 (39%) screened positive for ASD/PTSD symptoms, and 77/280 (27.5%) screened positive for both depressive and ASD/PTSD symptoms. Patients with facial burns were significantly more likely to have ASD/PTSD in both inpatient (14/29, p< 0.001) and outpatient (29/53, p=0.011) settings. Lower extremity burns were associated with depression as outpatients (64/148, p=0.049). Female outpatients were more likely to report depression (52/103 p=0.001) & ASD/PTSD (44/103, p=0.339) though ASD/PTSD was not statistically significant. For a number of patients, results varied between inpatient & outpatient screenings. About 35% of patients who screened negative for depression or ASD/PTSD as an inpatient then screened positive as an outpatient. Similarly, some patients screened positive for depression (23%) or ASD/PTSD (34%) as an inpatient and then screened negative as an outpatient.

**Conclusions:**

Mental health screening of individuals in our burn center yielded a significant number of positive screens for depression and ASD/PTSD symptoms, validating the need for screening of all burn patients. Comparisons of inpatient and outpatient screens for previously hospitalized patients showed changes in depression and PTSD symptoms over time. Positive screens resulted in referrals to community and mental health resources.

**Applicability of Research to Practice:**

Mental health screenings should be performed at multiple stages of recovery from burn injuries as manifestations of depression and ASD/PTSD may change over time.

